# Endobronchial Valves for Bronchoscopic Lung Volume Reduction in Severe Emphysema: A Reversible and Non-Surgical Treatment for Patients Who May or May Not Be Candidates for Lung Transplantation

**DOI:** 10.3390/diagnostics16111639

**Published:** 2026-05-27

**Authors:** Mateus Fernandes, David Eldeiry, Ali Musani

**Affiliations:** Department of Medicine, Division of Pulmonary, Critical Care, and Sleep Medicine, Lenox Hill Hospital, Northwell Health, New York, NY 10075, USA; deldeiry@northwell.edu (D.E.); amusani@northwell.edu (A.M.)

**Keywords:** bronchoscopic lung volume reduction, endobronchial valve, chronic obstructive lung disease, emphysema

## Abstract

Chronic obstructive pulmonary disease remains a leading cause of death worldwide, with emphysema contributing significantly to dyspnea, exercise limitation, and mortality. Bronchoscopic lung volume reduction (BLVR) using endobronchial valves (EBVs) has emerged as a minimally invasive, reversible alternative to lung volume reduction surgery for carefully selected patients with severe emphysema who remain symptomatic despite optimal medical therapy. EBVs are one-way valves placed bronchoscopically to achieve complete lobar occlusion, inducing atelectasis of the most diseased lung segments while allowing better ventilated parenchyma to expand, thereby improving respiratory mechanics and reducing hyperinflation. Landmark randomized controlled trials demonstrated that BLVR using EBVs produces significant improvements in forced expiratory volume in one second (FEV_1_), exercise capacity, and quality of life comparable to surgical lung volume reduction but with reduced morbidity and mortality. Critical to treatment success is meticulous patient selection based on emphysema distribution, absence of collateral ventilation, and appropriate physiologic parameters. Pneumothorax represents the most common serious complication, occurring in approximately 26% of patients, though paradoxically, it indicates successful lobar occlusion and predicts favorable long-term outcomes. As the most extensively studied BLVR, endobronchial valve therapy represents a cornerstone intervention for appropriately selected patients with severe emphysema.

## 1. Introduction

Within the past 5 years, chronic obstructive pulmonary disease (COPD) has remained among the top four causes of death worldwide [1,2]. COPD affects more than 400 million people globally, and in 2021, it accounted for 5% of all deaths worldwide [3]. The burden of COPD is projected to increase, with cases expected to reach nearly 600 million by 2050 [4].

Hyperinflation in COPD occurs due to loss of elastic recoil and expiratory flow obstruction, which are both driven by emphysematous parenchymal destruction and airways abnormalities such as mucous obstruction, airway edema, heightened bronchial tone, and wall remodeling [5,6]. Hyperinflation is associated with dyspnea, impaired exercise tolerance, and increased mortality [7,8,9,10]. Accordingly, attempts to reduce hyperinflation are of interest, which is challenging for patients already on maximum medical therapy [11,12].

As COPD phenotypes become better defined, trends in the management have shifted to personalized interventions [13,14,15]. Surgical and interventional treatments require careful patient selection based on symptom burden despite optimal medical treatment, structural features on computed tomography (CT) imaging, comorbidities, physiologic testing, and the overall risk-benefit profile to the individual patient.

There are several surgical and bronchoscopic procedures aimed at reducing hyperinflation, reducing mucous secretion, improving gas exchange, and enhancing functional capacity [2,16]. Airway-centered interventions, such as nitrogen cryospray, rheoplasty, and targeted lung denervation, remain under investigation [17,18]. Interventions targeting emphysema include bullectomy, large airway stenting, endobronchial coiling, thermal vapor ablation, instillation of lung sealants, endobronchial valve placement, and lung volume reduction surgery [16,19,20,21].

## 2. Advanced Interventions

Lung volume reduction surgery (LVRS) was pioneered in the 1950s, which at that time involved open thoracotomy with resection of the most emphysematous area of lung [22,23]. Early experiences were associated with high morbidity and mortality [24,25]. The National Emphysema Treatment Trial (NETT) was a landmark study that compared LVRS with optimal medical therapy to optimal medical therapy alone, and provided critical insights for patient selection [26,27]. Patients with upper lobe-predominant emphysema showed the greatest benefit in survival, forced expiratory volume in 1 s (FEV_1_), residual volume (RV), exercise capacity, and quality of life [2,23,26,28]. Patients with homogeneous emphysema, a diffusion capacity of the lungs for carbon monoxide (DLCO) of ≤20%, FEV_1_ ≤ 20%, and lower body mass index (BMI) had an increased mortality risk [2,23,26,29].

Bronchoscopic lung volume reduction (BLVR) was introduced as a less invasive approach to lung volume reduction [2,27,30]. The spectrum of BLVR includes endobronchial valve therapy (EBV), self-activating coils, lung sealants, airway bypass stents, and thermal ablative techniques. Although techniques for delivery vary, they share a physiologic objective to reduce hyperinflation and improve lung, chest wall, and respiratory muscle mechanics [2,12,31]. Endobronchial targeting of hyperinflation began in the early 2000s with the introduction of EBV [30]. At this time, EBV is the most well-studied and widely adopted BLVR technique [32,33,34,35,36,37,38,39].

There are limited prospective studies that compare outcomes in BLVR versus LRVS [40]. A prospective study in the United Kingdom randomizing patients to EBV or LVRS found similar outcomes in incremental shuttle walk distance, dyspnea, and FEV_1_ across both groups at follow-up [40]. A prospective review of a comprehensive lung volume reduction program found that EBV was associated with improved transplant-free survival compared to LVRS [41]. However, about half of those undergoing EBV required additional procedures, and a third eventually underwent LVRS [41].

Recent network meta-analyses have compared the efficacy and safety of lung volume reduction therapies [42,43]. An analysis of 25 RCTs found that LVRS demonstrated the largest improvements in FEV_1_, six-minute walk distance (6MWD), and St. George’s Respiratory Questionnaire score (SGRQ), but was associated with increased mid-term mortality [42]. Among bronchoscopic procedures, EBV showed the highest efficacy for FEV_1_ improvement, followed by endobronchial coils [42]. Similarly, an analysis of 26 RCTs assessed early and overall mortality [43]. Interestingly, lung volume reduction therapy was associated with higher mortality compared to standard medical care [43]. Consistent with the prior analysis, LVRS and EBV significantly improved FEV_1_, residual volume, and 6MWD, with LVRS showing more improvement but carrying the highest adverse event rates. These meta-analyses suggest that while LVRS offers the greatest functional gains, bronchoscopic approaches provide a relatively safer alternative with similar improvements in lung function, exercise capacity, and quality of life.

The decision between performing EBV versus more modern surgical techniques, such as robot-assisted thoracoscopic LVRS, depends on a myriad of factors, including the extent and pattern of emphysema on CT, the presence of collateral ventilation, regional availability of therapies, local proficiency, and shared decision making [2].

## 3. History of Bronchoscopic Lung Volume Reduction

As previously mentioned, bronchoscopic lung volume reduction involves multiple techniques aimed at occluding hyperinflated parenchyma and reducing lung volumes, restoring diaphragmatic function, and improving respiratory mechanics. The two commonest EBV systems include the Zephyr one-way EBV (PulmonX Co., Redwood City, CA, USA) and the Spiration valve system (Spiration Inc., Redmond, WA, USA) [44,45].

Emphasys was the first multicenter experience reporting the use of EBV [32]. Emphasys EBVs (Emphasys Medical [now PulmonX]) were deployed in 98 patients across seven countries. Patients with severe symptomatic heterogeneous emphysema despite maximal medical therapy were included. The target lobe was determined based on radiographic and ventilation/perfusion (V/Q) scan data. Improvements were seen in FEV_1_, forced vital capacity (FVC), RV, and exercise tolerance. In subgroup analysis, the greatest benefit was seen in patients with unilateral and lobar placement, and those with RV > 225%.

The VENT trial (2010) was a landmark multicenter randomized control trial that supported the safety and efficacy of EBVs [33]. In total, 492 patients were randomized in a 2:1 ratio to undergo EBV treatment versus optimized medical treatment. Patients in the treatment group had significant improvement in FEV_1_ and 6MWD. Subgroup analysis showed that the greatest benefit was seen in those with intact fissures and heterogeneous disease, which set the stage for future trials.

These insights from the VENT trial were further explored in the BeLieVer-HIFi (2015) and STELVIO (2015) trials [34,35]. BeLieVer-HIFi was a double-blind sham-controlled trial including 50 patients with heterogeneous emphysema and a target lobe with an intact interlobar fissure on CT. All patients underwent measurement of collateral ventilation using the Chartis (PulmonX Co., Redwood City, CA, USA) balloon catheter system, while target lobe selection was based on CT appearance. Patients with collateral ventilation detected on Chartis (despite intact fissures on CT) had no significant difference in FEV_1_, RV, 6MWD, or dyspnea-related quality of life measures compared to patients in the sham-bronchoscopy group. STELVIO was an open-label randomized controlled trial involving 68 patients with severe emphysema and confirmed absence of collateral ventilation (determined via Chartis). Patients in the intervention group had significantly greater increases in FEV_1_, FVC, and 6MWT distance at 6 months.

Overall, these two trials established that proper patient selection to exclude those with collateral ventilation was an important determinant of benefit [23]. These trials directly informed the study design for the subsequent TRANSFORM (2017) and LIBERATE (2018) trials, which then led to the Food and Drug Administration (FDA) approval of BLVR using EBV therapy [46,47].

TRANSFORM (2017) was a prospective multicenter randomized control trial involving 97 patients who were randomized in a 2:1 ratio to undergo BLVR via EBV versus standard of care [37]. The primary endpoint was improvement in FEV_1_ of ≥12%. More than half the patients in the EBV group achieved the primary outcome, in addition to significant improvements in 6MWD and dyspnea-related quality of life questionnaires. Results from TRANSFORM showed benefits comparable to those observed after LVRS, but with reduced morbidity [37,48].

LIBERATE (2018) was the largest multicenter randomized control trial evaluating the safety and efficacy of the Zephyr EBV [38]. A total of 190 patients with severe heterogeneous emphysema were randomized in a 2:1 fashion to receive Zephyr EBV versus standard of care, and followed for up to 12 months. The primary endpoint in LIBERATE was ≥15% FEV_1_ improvement. Nearly 50% of the intervention group met the primary endpoint, in addition to a significant improvement in 6MWD and dyspnea-related quality of life questionnaires.

**Figure 1:**
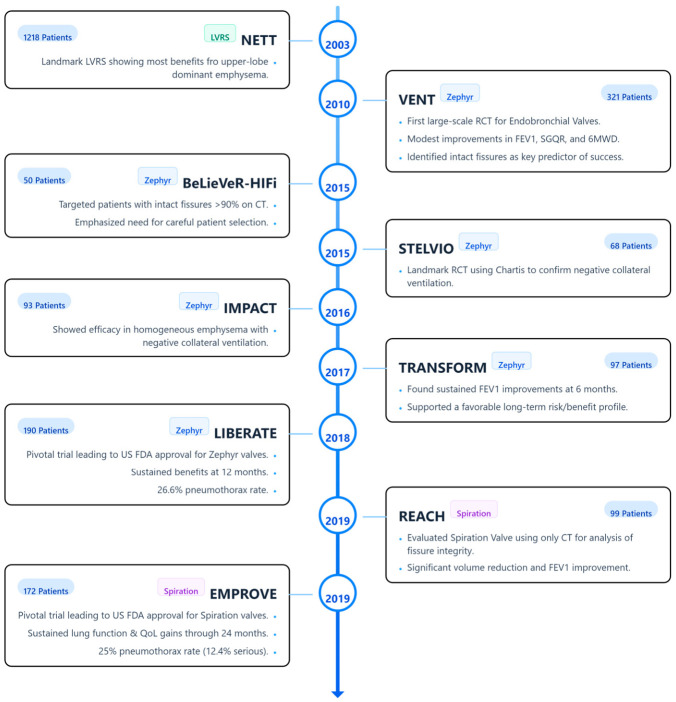
Flowchart showing key landmark trials for bronchoscopic lung volume reduction using endobronchial valves, over time.

REACH (2019) and EMPROVE (2019) were multicenter randomized controlled trials evaluating the Spiration Valve System in patients with severe emphysema [49,50]. Notably, patient selection relied solely on CT assessment of fissure integrity without intraprocedural collateral ventilation measurement. Both trials demonstrated significant improvements in FEV_1_ and most secondary endpoints. Long-term follow-up at 24 months in EMPROVE demonstrated sustained improvements in lung function, quality of life measures, and dyspnea [50]. Additional information about the results from trials discussed in this section is available in Table 1 and summarized in Figure 1.

Less frequent BLVR modalities, such as self-activating coils, targeted thermal ablation, and the instillation of lung sealants or airway bypass stents, are outside the scope of this review.

## 4. Pathophysiology

In emphysematous COPD, air trapping results from the combination of parenchymal destruction with loss of elastic recoil and expiratory flow obstruction from small airways disease. The loss of alveolar attachments to small airways and decreased elastic recoil prevent airways from remaining open during expiration [2]. This creates both static hyperinflation (increased resting lung volumes from emphysematous destruction) and dynamic hyperinflation (progressive air trapping during exercise when expiratory time is insufficient) [2,51,52].

Hyperinflation contributes to dyspnea through multiple mechanisms: it places the diaphragm at a mechanical disadvantage, increases the work of breathing, and creates a dissociation between respiratory effort and the ability to increase tidal volume [51,52]. This pathophysiology correlates with impaired exercise tolerance, increased hospitalizations, respiratory failure, and increased mortality [2,51].

As reviewed above, EBVs are one-way valves placed bronchoscopically in all segmental bronchi of a target lobe. The valves allow air to exit during expiration but prevent air from entering during inspiration [23]. Treatment aims to induce complete lobar occlusion, leading to deflation and absorption atelectasis of the treated lobe [23]. The resulting volume reduction decreases overall lung hyperinflation, allowing the ipsilateral non-targeted lobes to expand, which also improves respiratory mechanics by improving ventilation and perfusion matching in healthier lung, and restoring the diaphragm to a more favorable position to reduce the work of breathing [2,23,53].

The development of complete lobar atelectasis is associated with improved outcomes and survival [2,54]. One study found that none of the patients who developed atelectasis after BLVR died during follow-up, whereas 8 of 14 without atelectasis died (*p* = 0.026) [54]. Recent data suggest that improvements in exercise capacity and quality of life are independent predictors of survival, even more so than the presence of complete atelectasis itself [55]. There have been case reports of sublobar targeting with EBV with comparative results to total lobar atelectasis. Sublobar targeting may be appropriate in cases when there is low lung reserve and a higher risk for decompensation with complete lobar atelectasis. This approach has not been explored in prospective studies [56].

## 5. Fissure Completeness

**Figure 2:**
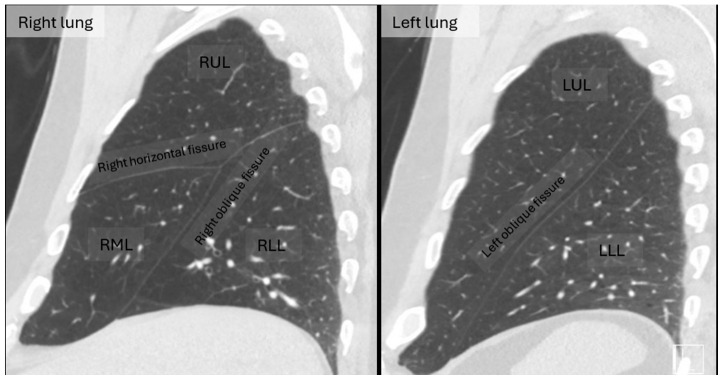
Chest computed tomography scans in sagittal plane demonstrating fissures assessed in the evaluation for endobronchial valve treatment. RUL: Right upper lobe. RML: Right middle lobe. RLL: Right lower lobe. LUL: Left upper lobe. LLL: Left lower lobe.

The success of EBV therapy critically depends on the absence of collateral ventilation between lobes, which requires intact interlobar fissures (Figure 2). When collateral channels exist, air can re-enter the occluded lobe, preventing effective volume reduction [23,35]. The following section briefly reviews fissure anatomy and methods for assessing completion.

The lungs are divided into five lobes by three interlobar fissures: the right oblique (major) fissure separating the right upper and middle lobes from the right lower lobe, the right horizontal (minor) fissure separating the right upper from the right middle lobe, and the left oblique (major) fissure separating the left upper from the left lower lobe [57]. Fissure completeness varies in both normal individuals and those with COPD. In healthy populations, complete fissures are present in approximately 72–77% for oblique fissures, 54% for the right horizontal fissure, and 72% for the left oblique fissure [58,59] on CT chest evaluation. Studies in individuals with COPD specifically show similar rates, with average fissure integrity of 80–82% for oblique fissures, 61–62% for the right horizontal fissure, and 81–83% for the left oblique fissure across all GOLD stages [59].

### 5.1. Visual Assessment

Fissure integrity assessment on CT requires careful attention to imaging technique and viewing planes. Visual assessment by radiologists using semi-quantitative scales (typically 5% increments) shows substantial interobserver agreement [60]. When compared to direct surgical assessment, CT overestimates completeness of the right minor fissure (positive predictive value of 33%) and underestimates completeness of the right major fissure (negative predictive value of 29%), though it performs moderately well for the left fissure (positive predictive value of 75%) [61]. Using the maximum intensity projection technique, thoracic radiologists achieve an accuracy of 76.8–85.1% for identifying complete fissures compared to surgical findings [62].

### 5.2. Quantitative Assessment

**Figure 3:**
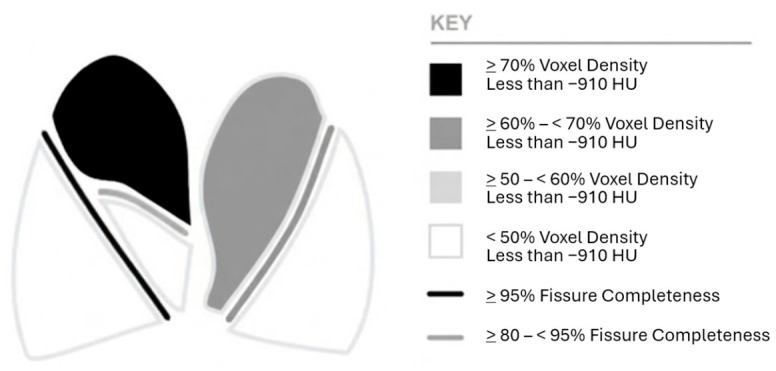
StratX report demonstrating fissure completeness and emphysema severity as quantified by voxel density.

Quantitative CT software tools have been developed to provide an objective, automated fissure completeness assessment. These include StratX (PulmonX, Redwood City, CA, USA), which was designed to assist in lung assessment for BLVR candidacy, and other platforms like Thirona (Nijmegen, The Netherlands) and VIDA Diagnostics (Coralville, IA, USA), which provide less specific assessments using artificial intelligence [63,64,65]. These tools use automated lung and lobe segmentation algorithms to calculate a fissure completeness score (expressed as the percentage of the lobar border defined by an intact fissure). For our discussion, we will focus on the StatX platform, which has been best studied in patients undergoing BLVR (Figure 3).

In addition to fissure integrity assessment, StratX provides lobar emphysema scores (also known as destruction score) calculated as the percentage of voxels with density ≤−910 or ≤−950 Hounsfield Units using automated lobar segmentation and densitometric analysis [23,44,53]. This allows for quantification of emphysema severity for each lobe, allowing identification of the most diseased target lobe and assessment of heterogeneity by comparing scores between lobes [23,53]. These voxel density measurements correlate with pathological emphysema, airflow obstruction, and clinical outcomes [38,44].

Beyond the lung parenchyma and fissures, V/Q single-photon emission computed tomography (SPECT) scans provide complementary functional data when multiple lobes are suitable targets, particularly in homogeneous emphysema [63,66]. Lobes with lower baseline perfusion are preferentially targeted, as these contribute less to gas exchange. Retrospective analysis of the VENT trial assessing perfusion showed that patients with low target lobe perfusion achieved greater improvement in 6MWD, independent of the degree of target lobe destruction [67,68]. Therefore, perfusion scans serve as a useful adjunct for BLVR planning when multiple target lobes are considered.

### 5.3. Bronchoscopic Assessment

In general, a fissure completeness score (FCS) > 95% indicates no significant collateral ventilation, making BLVR favorable [63,65]. The intermediate zone (FCS 80–95%) represents diagnostic uncertainty where quantitative CT alone is insufficient for treatment decisions [63,65].

As mentioned above, the Chartis system is a bronchoscopic tool that directly measures collateral ventilation in real-time [69,70]. During bronchoscopy, a balloon catheter is advanced through the working channel of a flexible bronchoscope and inflated to temporarily occlude the target lung segment [35,69]. The balloon blocks inspiratory flow while allowing expiratory flow through the catheter, which is measured by a console [35].

**Figure 4:**
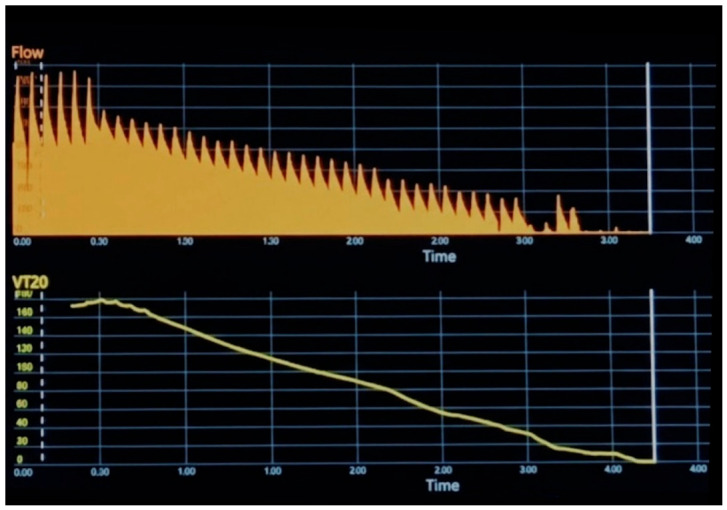
Chartis assessment showing flow gradually declining to zero, consistent with negative collateral ventilation.

Chartis interpretation is based on characteristic flow patterns. Continuous expiratory flow indicates collateral ventilation is present (CV+), while flow gradually declining to zero indicates absence of collateral ventilation (CV−) [35,69], as shown in Figure 4. Additional phenotypes include “low flow” (minimal airflow suggesting either very low collateral ventilation or poor catheter positioning) and “collapse phenomenon” (sudden cessation of flow due to airway collapse, more frequently seen in the lower lobes) [69,71].

## 6. Patient Selection

Candidates for BLVR should be clinically stable and on optimal medical therapy [44]. Clinical stability, generally defined as the absence of a COPD exacerbation for at least 6 to 8 weeks, is a prerequisite for proceeding with the intervention. Those with frequent, recurrent exacerbations are typically excluded. Optimal medical therapy should be confirmed before intervention, which includes guideline-directed inhaled therapy (typically a combination of long-acting β_2_-agonist, a long-acting muscarinic antagonist, and, in select cases, inhaled corticosteroids), as well as supplemental oxygen when indicated, smoking cessation, and appropriate vaccinations [2].

Participation in a structured pulmonary rehabilitation program is strongly recommended and was a prerequisite in several landmark trials [44]. Pulmonary rehabilitation optimizes functional capacity and ensures that residual dyspnea and exercise limitation are attributable to hyperinflation rather than deconditioning. It also establishes a reliable baseline from which post-procedural improvement can be assessed.

Candidates for endobronchial valve therapy must then meet clinical, physiological, and imaging criteria that identify those most likely to benefit from the procedure, as outlined in Table 2 [44,53].

Patients with COPD commonly have other comorbidities that may contribute to dyspnea or exercise limitation. Exclusion criteria identify patients with cardiac or pulmonary limitations that may not improve or may worsen with BLVR, as well as those with suspicious lung nodules requiring follow-up (Table 2) [66,67].

Cardiac contraindications (heart failure, pulmonary hypertension, uncontrolled arrhythmias) and structural lung abnormalities (such as severe bronchiectasis or interstitial lung disease) identify patients whose functional limitations and symptoms are not entirely due to emphysema [44,66,67,72]. Suspicious pulmonary nodules in the target lobe require satisfactory surveillance or tissue diagnosis before consideration for BLVR.

As noted, significant pulmonary hypertension is an exclusion criterion for BLVR. However, pulmonary artery systolic pressure (PASP) measured by echocardiography may be inaccurate. A retrospective review found that in those with suspected pulmonary hypertension based on echocardiography, 88% of patients did not have significant pulmonary hypertension as determined by right heart catheterization, and of these, nearly half (47%) qualified for BLVR [73]. Patients who otherwise meet BLVR criteria should be considered for confirmatory RHC before exclusion.

While rigorous criteria are essential for designing high-quality trials, there are subsets of patients who still benefit from BLVR despite not meeting all criteria [74]. A retrospective review compared patients who underwent BVLR meeting the LIBERATE trial criteria to those traditionally excluded due to higher FEV_1_ or 6MWD, or lower RV, and found that there were comparable improvements in FEV_1_, St. George’s Respiratory Questionnaire score (SGRQ), and 6MWD between both groups [75].

Ultimately, BLVR success relies on careful patient selection supported by several clinical trials and appropriate exclusion of patients whose symptoms are driven by comorbidities rather than emphysema, which may represent alternative targets for optimization.

## 7. Procedure Approach

BLVR can be performed with moderate sedation or general anesthesia [35,44]. While general anesthesia with endotracheal intubation is the most common approach in contemporary practice, moderate sedation remains a viable option and was used in 71.5% of patients in the VENT study [33,55]. A flexible bronchoscope with at least a 2.8 mm working channel to accommodate both the Chartis catheter and valve delivery systems is used [35]. Two endobronchial valve systems are currently available: the Zephyr valve and the Spiration valve, both demonstrating comparable efficacy in improving FEV_1_ and quality of life. The valves differ in design characteristics and deployment, and are available in multiple sizes to accommodate varying airway caliber.

**Figure 5:**
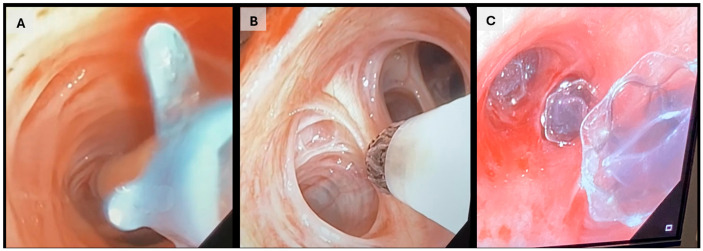
(**A**) Sizing tool used when placing a Zephyr valve. The short and long wings of the sizing tool correspond with specific valve sizes. (**B**) Deployment of the Zephyr valve, aligning the deployment tool to aim at the carina of the target segment. (**C**) Post-deployment view of valves.

The first step of the BLVR procedure is to confirm the absence of collateral ventilation in the target lobe using the Chartis system [35,44]. Next, valve size is determined by deploying a sizing tool with varying diameters (Figure 5) [44]. Valves should be deployed in all segments or subsegments of the target lobe to ensure complete occlusion, typically requiring placement of 3–4 valves per patient (Figure 5) [44]. Valve placement should be sequentially placed to avoid obstruction that would prevent delivery of subsequent valves, generally proceeding from distal to proximal segments [35,44].

Postoperatively, patients require close monitoring for at least 72 h, since most pneumothoraces occur within this period [2]. Chest radiography is performed immediately after the procedure and serially during the observation period [67]. Follow-up imaging at 4–6 weeks assesses for target lobe atelectasis and valve position, with consideration for valve repositioning if atelectasis has not occurred by 1 month [44].

## 8. Complications

### 8.1. Pneumothorax

Pneumothorax is the most common and serious complication of BLVR treatment, which occurs in approximately 26.6% of cases [2,76]. The majority (76%) develop within the first 72 h after valve placement, with a median time to pneumothorax of 7 h [2,77].

The mechanism underlying post-EBV pneumothorax involves rapid volume reduction in the treated lobe, causing expansion of the ipsilateral non-treated lobe, creating shearing forces that lead to pleural injury [78]. The time to atelectasis is crucial, with faster collapse leading to more abrupt expansion of adjacent lung tissue, increasing mechanical stress on the visceral pleura [78].

Pleural adhesions increase the risk for pneumothorax by creating fixed points that concentrate mechanical forces during atelectasis. Paraseptal emphysema also increases susceptibility to air leak and pneumothorax development [2,79]. Upper lobe treatment carries a higher pneumothorax risk, with an adjusted relative risk of 6.32 (95% CI 2.56–15.60) compared to lower lobe treatment [77].

The occurrence of pneumothorax paradoxically indicates successful target lobe occlusion and predicts better long-term outcomes [2,77]. Patients achieving complete lobar atelectasis, even those who developed pneumothorax, demonstrate greater improvements in lung function and reduced exacerbation rates compared to those with incomplete atelectasis [2].

### 8.2. Management of Pneumothorax Following BLVR

**Figure 6:**
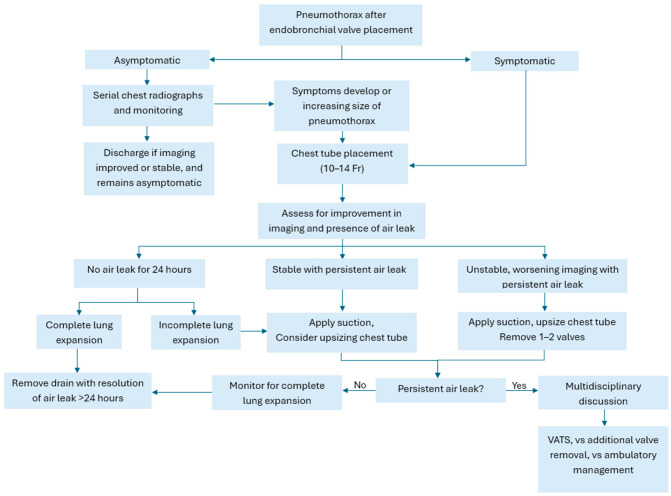
Algorithmic approach to pneumothorax post-endobronchial valve placement.

Timely management of pneumothorax following BLVR is essential, since patients undergoing BLVR have advanced disease with poor pulmonary reserve (Figure 6). A retrospective review assessing the management of 121 post-BLVR pneumothoraces found that 65% of cases were treated without valve removal. About half (49%) developed persistent air leak, requiring additional interventions such as Heimlich valve, additional chest tube placement, or valve removal [76].

For patients with an asymptomatic, small pneumothorax, inpatient observation with repeat chest radiography is recommended. Discharge may be considered if the pneumothorax is stable or improving on imaging and the patient remains clinically well. If imaging reveals an enlarging pneumothorax, chest tube insertion is required [78].

Symptomatic pneumothorax requires chest tube insertion. The chest tube may be removed if there has been no air leak for over 24 h, symptoms resolve, and the pneumothorax is improved on imaging [78].

If the symptoms worsen or there is a high-flow air leak with incomplete lung expansion, placing an additional chest tube or upsizing should be considered. In cases of persistent high air leak, defined as lasting more than 3 days, or when symptoms progress, then removal of one or two endobronchial valves may be performed [78].

Additional treatment options for prolonged air leak include surgery, use of a Heimlich valve, or pleurodesis. The timing and choice of treatment depend on the symptoms, patient preference, and the institution’s experience. Video-assisted thoracoscopic surgery (VATS) is an option for refractory persistent air leak. However, in BLVR candidates with severe heterogeneous emphysema, cardiopulmonary morbidity associated with surgery must be carefully considered. Most patients with valve-associated pneumothorax do not require surgical intervention and can be safely managed with chest tube drainage. For patients who are not surgical candidates, pleurodesis via talc slurry or autologous blood patch represents a viable alternative [78].

Discharge with a Heimlich valve is an option for clinically stable patients with persistent air leak, provided hospital care is easily accessible, and the patient has been adequately educated about drain care [78,80]. Systematic review data indicate that ambulatory management with Heimlich valves achieves outpatient treatment success rates of 77–86%, with few serious complications [80]. Ambulatory management allows for earlier discharge and has a relatively low rate of infection (2 out of 24 cases in one retrospective review) [81].

### 8.3. Other Complications

#### 8.3.1. Pneumonia

Pneumonia occurs in approximately 4–5% of patients within the first year, with half of the infections occurring in the treated lobe [38]. Most infections are managed medically, and pneumonia in the treated lobe unresponsive to oral and intravenous antibiotics may necessitate temporary valve removal [44].

#### 8.3.2. COPD Exacerbation

COPD exacerbations may occur in the early postprocedural period, with rates of 8–17% reported [35,38]. However, in the long term, patients achieve a reduction in exacerbation rates after EBV placement, with one study showing a decrease from 2.5 to 1.8 exacerbations per year, and another reporting an incidence rate ratio of 0.56 for severe exacerbations [82,83].

#### 8.3.3. Granulation Tissue Formation

Granulation tissue develops due to an inflammatory response to the valve, similar to any other foreign body [84]. In one study of valve removal after >6 months, granulation tissue was observed in 39.5% of patients [84]. Severe granulation tissue can cause valve malfunction, migration, post-obstructive pneumonia, or hemoptysis requiring valve removal [44,84]. Management typically includes systemic glucocorticoids, with additional local treatments such as cryotherapy or intralesional glucocorticoid injection as needed [44].

Despite these complications, BLVR demonstrates sustained clinical benefits [85]. Studies with 24-month follow-up show maintained improvements in lung function, quality of life, and dyspnea [85]. Importantly, BLVR is reversible, without long-term sequelae in most cases [44,84].

## 9. Revision of Endobronchial Valves

When counseling patients on BLVR, it is imperative to highlight that revision bronchoscopy may be required. Expert panel consensus recommends revision for patients without volume reduction 6 weeks after BLVR, or those who experience a loss of volume reduction, loss of treatment benefit, persistent cough or hemoptysis, or obstructive pneumonia [44]. Prior studies indicate that approximately 20% to 41% of patients require at least one revision bronchoscopy [86,87].

In a recent retrospective review of 148 patients who underwent BLVR, the most common indications for revision were loss of previously achieved atelectasis (67%) and failure to achieve initial atelectasis (28%) [87]. The most common cause for a loss of treatment effect was a paravalvular leak, occurring in 38% of cases. This may be due to incorrect valve sizing, suboptimal positioning, migration, or subsequent airway stretching that compromises the seal. Other etiologies include the formation of granulation tissue and mechanical valve malfunction. Notably, in 42% of revision procedures, no clear cause for the loss of atelectasis could be identified [87].

The probability of procedural success following revision may be predicted based on the patient’s initial response. Atelectasis may be re-established in 70% of cases [87]. In those who did not attain initial atelectasis, atelectasis may be achieved in 22% of cases [87]. The failure to achieve atelectasis may be due to inherent limitations of assessing for collateral ventilation, such as the presence of very slow collateral ventilation.

For the minority of cases not attaining atelectasis, patients may still derive subjective symptomatic benefit, such as improvement in SGRQ or 6MWD. This may be due to partial volume reduction and significant redirection of airflow away from the target lobe [87]. Other patients may remain severely limited by dyspnea. Further bronchoscopic refinements may be offered, such as upsizing or more distal placement of valves, or alternative treatment options can be pursued, such as LVRS or lung transplant.

In patients with a high residual burden of hyperinflation in the untreated lung, performing BLVR in the contralateral lung is an option [88]. Recently, a retrospective series suggested that bilateral BLVR is safe and may improve outcomes, particularly residual volume (52% improvement). Prospective and larger studies are needed to more accurately assess this approach [88].

## 10. Conclusions

BLVR using endobronchial valves has emerged as a minimally invasive, reversible therapy for patients with severe emphysema who remain symptomatic despite optimal medical management. By targeting hyperinflation and improving respiratory mechanics, BLVR offers significant improvements in lung function, exercise capacity, and quality of life that were previously only achievable through higher-risk surgical interventions. However, the success of BLVR depends on careful patient selection, specifically the identification of target lobes with intact fissures and the absence of collateral ventilation. As long-term data continue to demonstrate sustained benefits and improved survival, endobronchial valves are poised to remain a cornerstone of advanced COPD care.

## Figures and Tables

**Table 1:** Trials evaluating endobronchial valves for the treatment of advanced emphysema.

Trial	Sample Size	Results	Adverse Events
Emphasys [32] 2006	98	Changes from baseline at day 90: -FEV_1_: 10.7%; *p* < 0.001 -FVC: 9.0%; *p* 0.024 -RV: −4.9%; *p* 0.025 -6MWT: 37 m; *p* < 0.001	17% experienced COPD exacerbation within 90 days. 8 serious complications, including death (1), pneumothorax requiring intervention (3), and prolonged air leak (4).
VENT [33] 2010	321 (220 intervention, 101 control)	Changes from baseline at 6 months in the EBV group: -FEV_1_: 6.8%; *p* 0.005 -6MWT: 19 m; *p* 0.02 -SGRQ: −3.4; *p* 0.04	4.2% of patients in the EBV group developed post-obstructive pneumonia, 4.2% developed pneumothorax, 8% experienced COPD exacerbation requiring admission.
BeLieVeR-HIFi [34] 2015	50 (25 intervention, 25 control)	Changes from baseline at 3 months in the EBV group: -FEV_1_: 9%; *p* 0.03 -TLC: −3%; *p* 0.04 -RV: −7%; *p* 0.05 -SGRQ: −4; *p* 0.34 -6MWT: 25 m; *p* 0.01	Comparable rates of COPD exacerbation (*p* 0.42), pneumonia (*p* 0.49), and pneumothorax (*p* 1). 2 deaths in the EBV group.
STELVIO [35] 2015	68 (34 intervention, 34 control)	Changes from baseline at 6 months in the EBV group: -FEV_1_: 18%; *p* 0.001 -FVC: 14%; *p* 0.005 -SGRQ: −14.7; *p* < 0.001 -6MWT: 74 m; *p* < 0.001	Increased number of COPD exacerbations (*p* 0.67), pneumonia (*p* 1.0), and pneumothorax (*p* 0.02) in the EBV group. Overall higher number of total serious events (*p* < 0.001).
IMPACT [36] 2016	93 (43 intervention, 50 control)	Changes from baseline at 3 months in the EBV group: -FEV_1_: 0.12 L; *p* < 0.001 -RV: −0.48 L; *p* 0.0113 -6MWT: 40 m; *p* 0.002	Increased number of total respiratory events (*p* < 0.001), increased number of pneumothorax (*p* < 0.001) in the EBV group. Similar rates of COPD exacerbation.
TRANSFORM [37] 2017	97 (65 intervention, 32 control)	Changes from baseline at 6 months in the EBV group: -FEV_1_: 20.7%; *p* < 0.001 -RV: −0.67 L; *p* = 0.002 -6MWT: 79 m; *p* < 0.001 -SGRQ: −6.5; *p* 0.031	Increased number of respiratory-related adverse events (*p* < 0.001) in the EBV group. Significantly higher rate of pneumothorax in the EBV group, with similar rates of pneumonia and COPD exacerbation.
LIBERATE [38] 2018	190 (128 intervention, 62 control)	Changes from baseline at 12 months in the EBV group: -FEV_1_: 18%; *p* < 0.001 -RV: −0.52 L; *p* < 0.01 -6MWT: 39 m; *p* 0.002 -SGRQ: −7; *p* 0.004	Increased number of respiratory-related events (*p* < 0.001) in the EBV group. Significantly higher rates of pneumothorax in the EBV group, with similar rates of pneumonia and COPD exacerbation.
REACH [49] 2019	99 (66 intervention, 33 control)	Changes from baseline at 3 months in the EBV group: -FEV_1_: 0.1 L; *p* 0.001 -RV: −0.52 L; *p* 0.63 -6MWT: 27 m; *p* 0.126 -SGRQ: −8; *p* 0.05	33% of the intervention group had an adverse event, including COPD exacerbation (12%) and pneumothorax (6%). 12% of the control group experienced a COPD exacerbation.
EMPROVE [50] 2019	172 (113 intervention, 59 control)	Changes from baseline at 6 months in the EBV group: -FEV_1_: 0.1 L; PP 1.00 -RV: −0.4 L; PP 0.999 -6MWT: −4 m; PP 0.74 -SGRQ: −8; PP 1.00	Significantly more adverse effects in the EBV group, with a between-group difference of 19%. 12% increased incidence of serious pneumothorax in the EBV group.

**Table 2:** Inclusion and exclusion criteria for bronchoscopic lung volume reduction using endobronchial valves.

Inclusion Criteria	Exclusion Criteria
Emphysema confirmed by CT imaging BMI < 35 kg/m^2^ Stable disease without recent exacerbation RV ≥ 175% predicted (or ≥200% predicted if homogeneous emphysema) FEV_1_ 15–45% predicted TLC ≥ 100% predicted 6MWD of 100–500 m (or 150–500 m if homogeneous) Not actively smoking Target lobe with little to no collateral ventilation	Prior lung transplant, LVRS, median sternotomy, or lobectomy Congestive heart failure, uncontrolled cardiac arrhythmia, myocardial infarction, or stroke Known allergies to nitinol, nickel, titanium, or silicone Large bullae occupying ≥30% of either lung Contraindications for bronchoscopy Severe hypercapnia (PaCO_2_ ≥ 50 on room air) and/or severe hypoxemia (PaO_2_ ≤ 45 on room air) Uncontrolled pulmonary hypertension (PASP > 45 mm Hg) Significant bronchiectasis, substantial sputum production (greater than 4 tablespoons daily) Severe interstitial lung disease Lung nodules requiring surveillance in the target lobe

## Data Availability

No new data were created or analyzed in this study. Data sharing is not applicable to this article.

## Abbreviations

The following abbreviations are used in this manuscript:

6MWD	Six-Minute Walk Distance
6MWT	Six-Minute Walk Test
BLVR	Bronchoscopic Lung Volume Reduction
BMI	Body Mass Index
COPD	Chronic Obstructive Pulmonary Disease
CT	Computed Tomography
CV+	Collateral Ventilation positive
CV−	Collateral Ventilation negative
DALY	Disability-Adjusted Life Years
DLCO	Diffusion Capacity of the Lungs for Carbon Monoxide
EBV	Endobronchial Valve
FCS	Fissure Completeness Score
FDA	Food and Drug Administration
FEV1	Forced Expiratory Volume in 1 s
FVC	Forced Vital Capacity
GOLD	Global Initiative for Chronic Obstructive Lung Disease
LVRS	Lung Volume Reduction Surgery
mMRC	Modified Medical Research Council (dyspnea scale)
NETT	National Emphysema Treatment Trial
PaCO_2_	Partial Pressure of Carbon Dioxide
PaO_2_	Partial Pressure of Oxygen
PASP	Pulmonary Artery Systolic Pressure
PFT	Pulmonary Function Test
PP	Posterior probability
RHC	Right Heart Catheterization
RV	Residual Volume
SGRQ	St. George’s Respiratory Questionnaire score
SPECT	Single-Photon Emission Computed Tomography
TLC	Total Lung Capacity
USD	United States Dollars
V/Q	Ventilation/Perfusion
VATS	Video-Assisted Thoracoscopic Surgery

## References

[1] Halpin D.M.G., Celli B.R., Criner G.J., Frith P., Lopez Varela M.V., Salvi S., Vogelmeier C.F., Chen R., Mortimer K., Montes de Oca M. (2019). The GOLD Summit on chronic obstructive pulmonary disease in low- and middle-income countries. Int. J. Tuberc. Lung Dis..

[2] Venkatesan P. (2025). GOLD COPD report: 2026 update. Lancet Respir. Med..

[3] De Oca M.M., Perez-Padilla R., Celli B., Aaron S.D., Wehrmeister F.C., Amaral A.F.S., Mannino D., Zheng J., Salvi S., Obaseki D. (2025). The global burden of COPD: Epidemiology and effect of prevention strategies. Lancet Respir. Med..

[4] Mathers C.D., Loncar D. (2006). Projections of global mortality and burden of disease from 2002 to 2030. PLoS Med..

[5] Celli B., Fabbri L., Criner G., Martinez F.J., Mannino D., Vogelmeier C., Montes de Oca M., Papi A., Sin D.D., Han M.K. (2022). Definition and Nomenclature of Chronic Obstructive Pulmonary Disease: Time for Its Revision. Am. J. Respir. Crit. Care Med..

[6] Hyatt R.E. (1983). Expiratory flow limitation. J. Appl. Physiol..

[7] Tantucci C., Donati P., Nicosia F., Bertella E., Redolfi S., De Vecchi M., Corda L., Grassi V., Zulli R. (2008). Inspiratory capacity predicts mortality in patients with chronic obstructive pulmonary disease. Respir. Med..

[8] Ozgur E.S., Nayci S.A., Ozge C., Tasdelen B. (2012). An integrated index combined by dynamic hyperinflation and exercise capacity in the prediction of morbidity and mortality in COPD. Respir. Care.

[9] O’Donnell D.E., Webb K.A. (2008). The major limitation to exercise performance in COPD is dynamic hyperinflation. J. Appl. Physiol..

[10] O’Donnell D.E., Laveneziana P. (2007). Dyspnea and activity limitation in COPD: Mechanical factors. COPD J. Chronic Obstr. Pulm. Dis..

[11] Thomas M., Decramer M., O’Donnell D.E. (2013). No room to breathe: The importance of lung hyperinflation in COPD. Prim. Care Respir. J..

[12] Gompelmann D. (2025). Endoscopic Lung Volume Reduction: International Textbook of Interventional Pulmonology.

[13] Mirza S., Benzo R. (2017). Chronic Obstructive Pulmonary Disease Phenotypes: Implications for Care. Mayo Clin. Proc..

[14] Miravitlles M., Soler-Cataluna J.J., Calle M., Soriano J.B. (2013). Treatment of COPD by clinical phenotypes: Putting old evidence into clinical practice. Eur. Respir. J..

[15] Celli B.R., Wedzicha J.A. (2019). Update on Clinical Aspects of Chronic Obstructive Pulmonary Disease. N. Engl. J. Med..

[16] DeMarco B., MacRosty C.R. (2023). Bronchoscopic Management of COPD and Advances in Therapy. Life.

[17] Hartman J.E., Garner J.L., Shah P.L., Slebos D.J. (2021). New bronchoscopic treatment modalities for patients with chronic bronchitis. Eur. Respir. Rev..

[18] Sciurba F.C., Dransfield M.T., Kim V., Marchetti N., Comellas A., Hogarth D.K., Majid A. (2023). Bronchial rheoplasty for chronic bronchitis: 2-year results from a US feasibility study with RheOx. BMJ Open Respir. Res..

[19] Marchetti N., Duffy S., Criner G.J. (2020). Interventional Bronchoscopic Therapies for Chronic Obstructive Pulmonary Disease. Clin. Chest Med..

[20] Mineshita M., Slebos D.J. (2014). Bronchoscopic interventions for chronic obstructive pulmonary disease. Respirology.

[21] Tana A., Zhang C., DiBardino D., Orton C.M., Shah P.L. (2024). Bronchoscopic interventions for chronic bronchitis. Curr. Opin. Pulm. Med..

[22] Brantigan O.C., Mueller E. (1957). Surgical treatment of pulmonary emphysema. Am. Surg..

[23] Shah P.L., Herth F.J., van Geffen W.H., Deslee G., Slebos D.J. (2017). Lung volume reduction for emphysema. Lancet Respir. Med..

[24] Wouters E.F. (2004). Management of severe COPD. Lancet.

[25] Fein A.M. (1998). Lung volume reduction surgery: Answering the crucial questions. Chest.

[26] Fishman A., Fessler H., Martinez F., McKenna R.J., Naunheim K., Piantadosi S., Weinmann G., Wise R., National Emphysema Treatment Trial Research Group (2001). Patients at high risk of death after lung-volume-reduction surgery. N. Engl. J. Med..

[27] Criner G.J., Cordova F., Sternberg A.L., Martinez F.J. (2011). The National Emphysema Treatment Trial (NETT): Part I: Lessons learned about emphysema. Am. J. Respir. Crit. Care Med..

[28] Sanchez P.G., Kucharczuk J.C., Su S., Kaiser L.R., Cooper J.D. (2010). National Emphysema Treatment Trial redux: Accentuating the positive. J. Thorac. Cardiovasc. Surg..

[29] Greening N.J., Vaughan P., Oey I., Steiner M.C., Morgan M.D., Rathinam S., Waller D.A. (2017). Individualised risk in patients undergoing lung volume reduction surgery: The Glenfield BFG score. Eur. Respir. J..

[30] Marruchella A., Faverio P., Bonaiti G., Pesci A. (2018). History of lung volume reduction procedures. J. Thorac. Dis..

[31] Criner G.J., Cordova F., Sternberg A.L., Martinez F.J. (2011). The National Emphysema Treatment Trial (NETT) Part II: Lessons learned about lung volume reduction surgery. Am. J. Respir. Crit. Care Med..

[32] Wan I.Y., Toma T.P., Geddes D.M., Snell G., Williams T., Venuta F., Yim A.P. (2006). Bronchoscopic lung volume reduction for end-stage emphysema: Report on the first 98 patients. Chest.

[33] Sciurba F.C., Ernst A., Herth F.J., Strange C., Criner G.J., Marquette C.H., Kovitz K.L., Chiacchierini R.P., Goldin J., McLennan G. (2010). A randomized study of endobronchial valves for advanced emphysema. N. Engl. J. Med..

[34] Davey C., Zoumot Z., Jordan S., McNulty W.H., Carr D.H., Hind M.D., Hansell D.M., Rubens M.B., Banya W., Polkey M.I. (2015). Bronchoscopic lung volume reduction with endobronchial valves for patients with heterogeneous emphysema and intact interlobar fissures (the BeLieVeR-HIFi study): A randomised controlled trial. Lancet.

[35] Klooster K., ten Hacken N.H., Hartman J.E., Kerstjens H.A., van Rikxoort E.M., Slebos D.J. (2015). Endobronchial Valves for Emphysema without Interlobar Collateral Ventilation. N. Engl. J. Med..

[36] Valipour A., Slebos D.J., Herth F., Darwiche K., Wagner M., Ficker J.H., Petermann C., Hubner R.H., Stanzel F., Eberhardt R. (2016). Endobronchial Valve Therapy in Patients with Homogeneous Emphysema. Results from the IMPACT Study. Am. J. Respir. Crit. Care Med..

[37] Kemp S.V., Slebos D.J., Kirk A., Kornaszewska M., Carron K., Ek L., Broman G., Hillerdal G., Mal H., Pison C. (2017). A Multicenter Randomized Controlled Trial of Zephyr Endobronchial Valve Treatment in Heterogeneous Emphysema (TRANSFORM). Am. J. Respir. Crit. Care Med..

[38] Criner G.J., Sue R., Wright S., Dransfield M., Rivas-Perez H., Wiese T., Sciurba F.C., Shah P.L., Wahidi M.M., de Oliveira H.G. (2018). A Multicenter Randomized Controlled Trial of Zephyr Endobronchial Valve Treatment in Heterogeneous Emphysema (LIBERATE). Am. J. Respir. Crit. Care Med..

[39] Ceulemans L.J., Vandervelde C.M., Everaerts S., Dooms C., Geysen H., Fontaine M., Bouneb S., Neyrinck A., De Leyn P., Janssens W. (2026). Low mortality and favourable outcome in lung volume reduction surgery for different emphysema morphologies. Eur. Respir. J..

[40] Buttery S.C., Banya W., Bilancia R., Boyd E., Buckley J., Greening N.J., Housley K., Jordan S., Kemp S.V., Kirk A.J.B. (2023). Lung volume reduction surgery versus endobronchial valves: A randomised controlled trial. Eur. Respir. J..

[41] Janssens W., Everaerts S., Filipow N., Vandervelde C., Coolen J., Geysen H., Blondeel A., Gyselinck I., Stylemans D., Niesten I. (2025). A Comprehensive Approach to Lung Volume Reduction Encompassing Surgical and Endobronchial Treatment of Severe Emphysema. Chest Pulm..

[42] Yamamoto S., Horita N., Imai R., Niitsu T. (2025). Surgical and Bronchoscopic Lung Volume Reduction for Severe Emphysema: A Systematic Review and Network Meta-analysis. Lung.

[43] Bo L., He X., Chen Y., Shi L., Li C. (2025). Lung Volume Reduction Therapies in Patients with Emphysema: A Systematic Review and Network Meta-Analysis. COPD J. Chronic Obstr. Pulm. Dis..

[44] Slebos D.J., Shah P.L., Herth F.J., Valipour A. (2017). Endobronchial Valves for Endoscopic Lung Volume Reduction: Best Practice Recommendations from Expert Panel on Endoscopic Lung Volume Reduction. Respiration.

[45] Sterman D.H., Mehta A.C., Wood D.E., Mathur P.N., McKenna R.J., Ost D.E., Truwit J.D., Diaz P., Wahidi M.M., Cerfolio R. (2010). A multicenter pilot study of a bronchial valve for the treatment of severe emphysema. Respiration.

[46] Herth F.J.F., Slebos D.J., Criner G.J., Valipour A., Sciurba F., Shah P.L. (2019). Endoscopic Lung Volume Reduction: An Expert Panel Recommendation—Update 2019. Respiration.

[47] Hartman J.E., Vanfleteren L., van Rikxoort E.M., Klooster K., Slebos D.J. (2019). Endobronchial valves for severe emphysema. Eur. Respir. Rev..

[48] Washko G.R., Fan V.S., Ramsey S.D., Mohsenifar Z., Martinez F., Make B.J., Sciurba F.C., Criner G.J., Minai O., Decamp M.M. (2008). The effect of lung volume reduction surgery on chronic obstructive pulmonary disease exacerbations. Am. J. Respir. Crit. Care Med..

[49] Li S., Wang G., Wang C., Gao X., Jin F., Yang H., Han B., Zhou R., Chen C., Chen L. (2019). The REACH Trial: A Randomized Controlled Trial Assessing the Safety and Effectiveness of the Spiration(R) Valve System in the Treatment of Severe Emphysema. Respiration.

[50] Criner G.J., Delage A., Voelker K., Hogarth D.K., Majid A., Zgoda M., Lazarus D.R., Casal R., Benzaquen S.B., Holladay R.C. (2019). Improving Lung Function in Severe Heterogenous Emphysema with the Spiration Valve System (EMPROVE). A Multicenter, Open-Label Randomized Controlled Clinical Trial. Am. J. Respir. Crit. Care Med..

[51] Rossi A., Aisanov Z., Avdeev S., Di Maria G., Donner C.F., Izquierdo J.L., Roche N., Similowski T., Watz H., Worth H. (2015). Mechanisms, assessment and therapeutic implications of lung hyperinflation in COPD. Respir. Med..

[52] Soffler M.I., Hayes M.M., Schwartzstein R.M. (2017). Respiratory Sensations in Dynamic Hyperinflation: Physiological and Clinical Applications. Respir. Care.

[53] Klooster K., Slebos D.J. (2021). Endobronchial Valves for the Treatment of Advanced Emphysema. Chest.

[54] Hopkinson N.S., Kemp S.V., Toma T.P., Hansell D.M., Geddes D.M., Shah P.L., Polkey M.I. (2011). Atelectasis and survival after bronchoscopic lung volume reduction for COPD. Eur. Respir. J..

[55] Roodenburg S.A., Slebos D.J., van Dijk M., Koster T.D., Klooster K., Hartman J.E. (2023). Improved exercise capacity results in a survival benefit after endobronchial valve treatment. Respir. Med..

[56] Asghar A., Forth V., Shafiq M. (2024). Using Sub-lobar Bronchoscopic Lung Volume Reduction to Optimize Safety and Efficacy in a Case of High-risk Emphysema. J. Bronchol. Interv. Pulmonol..

[57] Van der Molen M.C., Hartman J.E., Vermeulen C.J., van den Berge M., Faiz A., Kerstjens H.A.M., Charbonnier J.P., Vanfleteren L., Slebos D.J. (2021). Determinants of Lung Fissure Completeness. Am. J. Respir. Crit. Care Med..

[58] Abdu S.M., Ali S.Y., Assefa E.M., Muhaba E.S. (2026). Anatomical Variations of the Lung Lobes and Fissures: A Systematic Review and Meta-Analysis. Clin. Anat..

[59] Pu J., Wang Z., Gu S., Fuhrman C., Leader J.K., Meng X., Tedrow J., Sciurba F.C. (2014). Pulmonary fissure integrity and collateral ventilation in COPD patients. PLoS ONE.

[60] Koenigkam-Santos M., de Paula W.D., Owsijewitsch M., Wielputz M.O., Gompelmann D., Schlemmer H.P., Kauczor H.U., Heussel C.P., Puderbach M. (2013). Incomplete pulmonary fissures evaluated by volumetric thin-section CT: Semi-quantitative evaluation for small fissure gaps identification, description of prevalence and severity of fissural defects. Eur. J. Radiol..

[61] Kent M.S., Ridge C., O’Dell D., Lo P., Whyte R., Gangadharan S.P. (2015). The accuracy of computed tomography to predict completeness of pulmonary fissures. A prospective study. Ann. Am. Thorac. Soc..

[62] Soder S.A., Perin F.A., Felicetti J.C., Camargo J.J.P., Camargo S.M., Hochhegger B., Teixeira P.J.Z. (2022). Accuracy of multidetector computed tomography with maximum intensity projection technique in the assessment of fissure integrity. J. Thorac. Dis..

[63] Klooster K., Koster T.D., Ruwwe-Glosenkamp C., Theilig D., Doellinger F., Saccomanno J., Kerstjens H.A.M., Slebos D.J., Hubner R.H. (2020). An Integrative Approach of the Fissure Completeness Score and Chartis Assessment in Endobronchial Valve Treatment for Emphysema. Int. J. Chron. Obstr. Pulm. Dis..

[64] Schuhmann M., Raffy P., Yin Y., Gompelmann D., Oguz I., Eberhardt R., Hornberg D., Heussel C.P., Wood S., Herth F.J. (2015). Computed tomography predictors of response to endobronchial valve lung reduction treatment. Comparison with Chartis. Am. J. Respir. Crit. Care Med..

[65] Koster T.D., van Rikxoort E.M., Huebner R.H., Doellinger F., Klooster K., Charbonnier J.P., Radhakrishnan S., Herth F.J., Slebos D.J. (2016). Predicting Lung Volume Reduction after Endobronchial Valve Therapy Is Maximized Using a Combination of Diagnostic Tools. Respiration.

[66] Martin M.J., Dulohery Scrodin M.M., Edell E.S., Aguirre E.A., Rajagopalan S., Bartholmai B.J., Peikert T. (2023). Bronchoscopic Lung Volume Reduction: Highlighting the Patient Selection Process. Mayo Clin. Proc..

[67] Gupta Y.S., Simpson S., Graham R., Kumaran M., Dako F., Hota P., Dass C. (2025). Imaging of Bronchoscopic Lung Volume Reduction Using Endobronchial Valves. Radiographics.

[68] Argula R.G., Strange C., Ramakrishnan V., Goldin J. (2013). Baseline regional perfusion impacts exercise response to endobronchial valve therapy in advanced pulmonary emphysema. Chest.

[69] Saccomanno J., Hubner R.H., Witzenrath M., Doellinger F., Dittrich A.S., Kontogianni K., Herth F., Brock J.M. (2023). Bronchoscopic Measurement of Collateral Ventilation: State of the Art. Respiration.

[70] Herth F.J., Eberhardt R., Gompelmann D., Ficker J.H., Wagner M., Ek L., Schmidt B., Slebos D.J. (2013). Radiological and clinical outcomes of using Chartis to plan endobronchial valve treatment. Eur. Respir. J..

[71] Gesierich W., Samitas K., Reichenberger F., Behr J. (2016). Collapse phenomenon during Chartis collateral ventilation assessment. Eur. Respir. J..

[72] Zantah M., Gangemi A.J., Criner G.J. (2020). Bronchoscopic lung volume reduction: Status quo. Ann. Transl. Med..

[73] Mahajan A.K., Collar N., Muldowney F., Gorka P., Duong D.K., Patel P.P., Cicenia J., Hogarth D.K., Nathan S. (2025). Echocardiography Findings for Pulmonary Hypertension During Workup for Bronchoscopic Lung Volume Reduction. J. Bronchol. Interv. Pulmonol..

[74] Pu C.Y., Kheir F. (2024). Is It Time to Expand the Criteria for Bronchoscopic Lung Volume Reduction in Very Low FEV1 and DLCO Emphysema. J. Bronchol. Interv. Pulmonol..

[75] Mahajan A.K., Patel P.P., Duong D.K., Shah N., Collar N., Muldowney F., Ochoa Cuba M.A., Wang H., Subramanian M., Suzuki K. (2026). Outcomes From Expanded Qualification Parameters for Bronchoscopic Lung Volume Reduction (BLVR). J. Bronchol. Interv. Pulmonol..

[76] Low S.W., Swanson K.L., Lee J.Z., Tan M.C., Cartin-Ceba R., Sakata K.K., Maldonado F. (2022). Complications of Endobronchial Valve Placement for Bronchoscopic Lung Volume Reduction: Insights From the Food and Drug Administration Manufacturer and User Facility Device Experience (MAUDE). J. Bronchol. Interv. Pulmonol..

[77] Jorgensen K.H., Christensen T.D., Titlestad I.L., Davidsen J.R., Bock K., Jensen K., Bendixen M., Jorgensen O.D., Perch M., Bendstrup E. (2025). Risk Factors for Pneumothorax After Treatment With Endobronchial Valves: A Cohort Study. Respirology.

[78] Van Dijk M., Sue R., Criner G.J., Gompelmann D., Herth F.J.F., Hogarth D.K., Klooster K., Kocks J.W.H., de Oliveira H.G., Shah P.L. (2021). Expert Statement: Pneumothorax Associated with One-Way Valve Therapy for Emphysema: 2020 Update. Respiration.

[79] Gompelmann D., Lim H.J., Eberhardt R., Gerovasili V., Herth F.J., Heussel C.P., Eichinger M. (2016). Predictors of pneumothorax following endoscopic valve therapy in patients with severe emphysema. Int. J. Chron. Obstr. Pulm. Dis..

[80] Brims F.J., Maskell N.A. (2013). Ambulatory treatment in the management of pneumothorax: A systematic review of the literature. Thorax.

[81] Mahajan A.B., Bari M., Collar N., Chakravorty S., Duong D.K., Suzuki K., Patel P.P., Weyant M.J., Hogarth D.K. (2024). Safety of Home Discharge With a Chest Tube After Bronchoscopic Lung Volume Reduction Complicated by Persistent Airleak. J. Bronchol. Interv. Pulmonol..

[82] Brock J.M., Bohmker F., Schuster P.U., Eberhardt R., Gompelmann D., Kontogianni K., Dittrich S., Benjamin N., Herth F. (2023). Endobronchial lung volume reduction with valves reduces exacerbations in severe emphysema patients. Respir. Med..

[83] Borg M., Ibsen R., Hilberg O., Lokke A. (2025). Real-Life Nationwide Outcomes of Bronchoscopic Lung Volume Reduction with Endobronchial Valves in Severe Chronic Obstructive Pulmonary Disease. Respiration.

[84] Gompelmann D., Gerovasili V., Kontogianni K., Schuhmann M., Eberhardt R., Herth F.J.F., Polke M. (2018). Endoscopic Valve Removal >180 Days since Implantation in Patients with Severe Emphysema. Respiration.

[85] Criner G.J., Mallea J.M., Abu-Hijleh M., Sachdeva A., Kalhan R., Hergott C.A., Lazarus D.R., Mularski R.A., Calero K., Reed M.F. (2024). Sustained Clinical Benefits of Spiration Valve System in Patients with Severe Emphysema: 24-Month Follow-Up of EMPROVE. Ann. Am. Thorac. Soc..

[86] Roodenburg S.A., Klooster K., Hartman J.E., Koster T.D., van Dijk M., Slebos D.J. (2021). Revision Bronchoscopy After Endobronchial Valve Treatment for Emphysema: Indications, Findings and Outcomes. Int. J. Chron. Obstr. Pulm. Dis..

[87] Mahajan A.K., Collar N., Muldowney F., Patel P.P., Hogarth D.K., Duong D.K. (2025). Incidence and Outcomes of Revision Bronchoscopies Following Bronchoscopic Lung Volume Reduction (BLVR). J. Bronchol. Interv. Pulmonol..

[88] Asghar A., Fonseca-Paricio M., Ravikumar N., Hammer M.M., Forth V., Wagh A., Hogarth D.K., Shafiq M. (2026). Sequential Bilateral Bronchoscopic Lung Volume Reduction for Residual Hyperinflation Following Successful Index Procedure in Pulmonary Emphysema: A Multicenter Study. J. Bronchol. Interv. Pulmonol..

